# The Emerging Role of MicroRNAs in Nasal Inflammatory Diseases and Tumors: From Bench to Bedside

**DOI:** 10.3390/genes16030295

**Published:** 2025-02-28

**Authors:** Antonella Loperfido, Carlo Cavaliere, Bruno Fionda, Simonetta Masieri, Gianluca Bellocchi, Massimo Re, Marco Tomasetti

**Affiliations:** 1 Otolaryngology Unit, San Camillo Forlanini Hospital, Circonvallazione Gianicolense 87, 00152 Rome, Italy; 2 Department of Sense Organs, Sapienza University, Piazzale Aldo Moro 5, 00185 Rome, Italy; 3 Fondazione Policlinico Universitario Agostino Gemelli IRCCS, 00168 Rome, Italy; 4 Department of Oral and Maxillofacial Sciences, Sapienza University, Piazzale Aldo Moro 5, 00185 Rome, Italy; 5 Department of Clinical and Molecular Sciences, Polytechnic University of Marche, Via Tronto 10/a, 60020 Ancona, Italy

**Keywords:** microRNAs, miRNAs, chronic rhinosinusitis, allergic rhinitis, sinonasal tumors, biomarkers, miRNA therapy, personalized medicine

## Abstract

Background/Objectives: MicroRNAs (miRNAs) play a crucial role in regulating immune responses and have been implicated in the pathogenesis of various nasal diseases, including chronic rhinosinusitis (CRS), allergic rhinitis (AR), and sinonasal tumors. This review comprehensively explores the emerging role of miRNAs in inflammatory and oncological nasal diseases, highlighting their diagnostic, prognostic, and therapeutic potential. Methods: A comprehensive review of the literature was conducted to summarize current findings on miRNA expression in nasal inflammatory conditions and tumors. Key studies evaluating miRNA-mediated regulatory mechanisms, potential biomarker applications, and therapeutic approaches were analyzed. Results: Altered miRNA expression profiles contribute to the pathogenesis of CRS, AR, and sinonasal tumors. Specific miRNAs, such as miR-125b and miR-155 are upregulated in CRS and AR, promoting inflammation and tissue remodeling. In sinonasal tumors, dysregulated miRNAs, including miR-126 and miR-34/miR-449 clusters, influence tumor progression and therapeutic response. Exosome-mediated miRNA delivery emerges as a promising avenue for precision medicine, offering novel strategies for miRNA-based diagnostics and therapies. Conclusions: miRNAs are key regulators of nasal diseases, with potential applications in non-invasive diagnostics and targeted therapies. Further research into miRNA-based interventions may improve treatment outcomes and contribute to the development of personalized medicine approaches for nasal inflammatory disorders and malignancies.

## 1. Introduction

Studies on microRNAs (miRNAs), a class of small non-coding RNA molecules consisting of approximately 18–25 nucleotides, began in the early 1990s with the work of the Ambros and Ruvkun groups in *Caenorhabditis elegans* [1,2]. These studies highlighted the pivotal role of miRNAs in post-transcriptional gene regulation, revolutionizing the discipline of molecular biology. This emerging field has since advanced through additional research that has demonstrated the functional roles of miRNAs in various biological processes, including development, differentiation, and apoptosis. Furthermore, not only do miRNAs play a crucial role in a variety of biological processes but also their aberrant expression is associated with the pathogenesis of numerous human diseases [3,4].

miRNAs play an essential role in modulating inflammation by regulating the expression of genes involved in immune responses [5]. Depending on the specific miRNA and its target genes, they can act either to stimulate or suppress inflammatory processes. Exploring the role of miRNAs in inflammatory conditions offers critical insights into their biogenesis pathways and highlights potential therapeutic strategies for various diseases [6], including those affecting the nose and paranasal sinuses.

Additionally, miRNAs are released into extracellular fluids, and extracellular miRNAs have been extensively studied as potential biomarkers for various diseases. Moreover, they function as signaling molecules, mediating cell-to-cell communication [7,8,9]. Interestingly, extracellular miRNAs have been reported as potential biomarkers due to their detectability in plasma, blood, urine, and other body fluids, even after years of sample storage [10]. In this regard, alterations in circulating miRNA levels have been linked to a broad spectrum of diseases, including cancer, cardiovascular diseases, neurodegenerative disorders, type 2 diabetes, obesity, and more [11,12,13,14,15]. Furthermore, alterations in miRNA levels have been demonstrated to correlate with variations in lifestyle factors, such as exercise, diet, and gut microbiota composition [16,17]. This suggests that extracellular miRNAs may serve as circulating indicators of an individual’s physiological state and as tools for precision medicine.

In addition to functioning as diagnostic biomarkers, miRNAs are emerging as promising therapeutic targets. Numerous studies emphasize their role as key regulators in various diseases, with extensive research exploring their therapeutic potential for challenging conditions [18,19].

The therapeutic potential of miRNAs in cancer is supported by their ability to regulate multiple genes involved in various signaling pathways. Compared to other gene therapy approaches, miRNAs exhibit greater advancement and biosafety. Exosomes exclusively modulate the post-transcriptional regulation of target genes, thereby minimizing the risk of tumorigenesis associated with off-target gene integration or unintended immune responses [20].

Although miRNA therapy is a rapidly developing field [21], clinical application remains a significant challenge due to issues with efficacy, specificity, and delivery in humans. In addition, exosomes have gained significant attention as efficient carriers for miRNA, as they are naturally packed and commonly transported by exosomes in normal and pathological cell–cell communication. Exosome are small vesicles ranging in size from 30 to 150 nm in diameter; they are derived from the late endosomal route/multivesicular body (MVB) and are secreted by two different mechanisms, constitutive release via the Trans–Golgi network and inducible release [22].

Concerning the nasal cavities and paranasal sinuses, miRNAs have been extensively investigated for their roles in both inflammatory disorders, such as chronic rhinosinusitis (CRS) and allergic rhinitis (AR), as well as in the pathogenesis and progression of oncological conditions affecting this area. These include benign tumors, such as sinonasal inverted papilloma and juvenile angiofibroma, and malignant tumors, such as sinonasal squamous cell carcinoma (SNSCC).

Table 1 summarizes the main findings of the original articles included in this review.

## 2. miRNAs in Inflammatory Nasal Diseases

### 2.1. Chronic Rhinosinusitis (CRS)

A key aspect introduced by the European Position Paper on Rhinosinusitis and Nasal (EPOS 2020) guidelines is the CRS endotype classification, which relies on specific pathophysiological mechanisms and molecular biomarkers. This classification identifies two dominant endotypes based on underlying immunological pathophysiology: type 2, primarily associated with the Th2 immune response, and non-type 2. The type 2 immune pathway, the most common in CRSwNP among Caucasians, is characterized by the overproduction of cytokines interleukin (IL)-4, IL-5, and IL-13, along with increased IgE levels and eosinophils [55,56,57,58]. The non-type 2 immune pathway involves a combination of type 1 and type 3 immune responses and is typically associated with significant neutrophil infiltration. Within these pathways, epithelial activation in response to environmental stimuli triggers dendritic cells, which subsequently drive the differentiation of Th1 and Th17 cells, resulting in non-eosinophilic inflammation. In particular, type 1 inflammation is mediated by T helper 1 (Th1) cells and ILC1, which activate mononuclear phagocytes. These cells produce interferon γ (IFN-γ), a key cytokine in type 1 inflammation. IFN-γ induces apoptosis of nasal epithelial cells and enhances neutrophil activity, playing a central role in the immune response to viral infections. In contrast, type 3 inflammation is triggered by extracellular bacteria and fungi, prompting epithelial cells to release osteopontin. This, in turn, activates dendritic cells to promote the differentiation of Th17 cells, which secrete the cytokines IL-17, IL-22, and IL-23 [59]. Generally, the non-type 2 immune pathway influences CRSsNP, which is associated with fibrosis and Th1 inflammation, and CRSwNP in Asians, as it exhibits a predominant Th1/Th17 pattern of inflammation [23].

When epithelial damage occurs, whether triggered by bacteria, allergens, viruses, or fungi, submucosal tissue can be exposed to external stimuli, promoting cytokine and chemokine production. Epithelial disruption enhances the immune response, and if the stimulus is sufficiently strong, it can trigger an acquired immune response [60]. The literature has extensively demonstrated that the pathogenesis of CRS is influenced by both genetic and environmental factors. The intricate interplay between multiple genetic loci and various environmental exposures likely explains the broad spectrum of molecular and clinical manifestations of CRS, with variable degrees of tissue inflammation and clinical symptoms, shaped by specific genetic and epigenetic variations. Of these factors, miRNAs are acknowledged to play a significant role in modulating gene expression and influencing cytokine-related functional outcomes [61]. In this regard, evidence suggests that miRNAs are involved in various inflammatory responses, either by regulating the intensity of inflammation or modulating the profile of recruited inflammatory cells [62].

In CRSwNP, the aberrant expression of miRNAs is associated with the activation of pro-inflammatory pathways, leading to inflammation and fibrosis, which subsequently contribute to the formation of nasal polyps [24,25]. Among these, miR-125b is one of the most extensively studied miRNAs in CRSwNP, as its expression is significantly elevated in nasal polyps compared to normal nasal mucosa, with even higher levels observed in eosinophilic CRSwNP (ECRSwNP) [26,27]. Its upregulation in CRS, particularly in ECRSwNP, suggests a potential link between miR-125b and Th2-driven inflammation [28,63,64]. Moreover, elevated serum levels of miR-125b have been detected in patients with bronchial asthma, a condition also characterized by type 2 inflammation and frequently associated with CRSwNP as a comorbidity [65]. Some authors propose that miR-125b could serve as a potential biomarker for CRSwNP and holds promise as a future therapeutic target [29].

Reports have also shown that miR-155 is upregulated in CRS, particularly in ECRSwNP [30]. Silveira et al. further identified increased levels of miRNA-205-5p in CRSwNP, especially in patients with higher type 2 inflammation, as indicated by elevated IL-5 levels, local eosinophilia, and more severe clinical presentations. They concluded that this miRNA may represent a promising target for further investigation in CRSwNP patients [23].

Distinct miRNAs have been identified across different CRSwNP subtypes. For instance, Bu et al. reported that miR-221 is upregulated in CRSwNP and may be specifically involved in regulating the cell cycle, apoptosis, and inflammation. Additionally, the authors found that miR-449a expression is higher in non-eosinophilic CRSwNP (non-ECRSwNP) samples compared to both ECRSwNP samples and healthy controls. They concluded that ECRSwNP and non-ECRSwNP patients exhibit distinct miRNA-mRNA regulatory networks, which may provide promising targets for the development of novel therapeutic approaches for managing CRSwNP [31].

In addition to upregulated miRNAs, numerous studies investigating miRNAs in CRS have identified a substantial number of downregulated miRNAs. For example, Li and Liu identified 207 miRNAs with low expression in ECRSwNP [66]. Similarly, Liu et al. reported decreased levels of miR-124 in CRSwNP [32], while Korde et al. demonstrated an inverse correlation between miR-1 expression and the degree of sinonasal eosinophilia in CRS patients [33].

Research on miRNAs in inflammatory airway diseases is rapidly advancing, with a growing number of studies emphasizing their role in regulating the immune response in CRS. For instance, miR-142-3p has been shown to amplify inflammation by interacting with cytokine pathways, including TNF-α [34]. Other authors have reported that miR-19a suppresses IL-10, a pleiotropic cytokine with elevated levels known to play a crucial role in the pathogenesis of CRSwNP [67]. Furthermore, miR-19a has been suggested as a novel therapeutic target for the treatment of allergic diseases [35].

Among the various miRNAs, miR-21-5p has been identified as one of the most conserved and abundant miRNAs detected in the nasal mucosa. In particular, it is recognized as an inflammamiR due to its role in inflammation and an oncomiR for its oncogenic activity. In this context, Luan et al. demonstrated that miR-21-5p promotes and exacerbates mucosal type 2 inflammation in CRSwNP by regulating the glucagon-like peptide-1 receptor/IL-33 signaling pathway [36].

Finally, several studies have demonstrated that miRNAs play a key role not only in the progression of inflammation but also in the tissue remodeling that characterizes CRSwNP. In this context, miR-155-5p and miR-21 have been shown to promote tissue remodeling through epithelial–mesenchymal transition (EMT), contributing to the formation of nasal polyps [37,38].

The pathological mechanisms of CRSwNP are poorly understood, and the high rate of recurrence poses a significant clinical challenge due to its high tissue heterogeneity. By analyzing the serum exosomal miRNA profiles in CRSwNP patients, it was found that exosomal miR-3174 and miR-192-3p are differently expressed in recurrent CRSwNP patients when compared with non-recurrent cases [68]. As well, serum exosomal miR-141-3p and miR-3679-5p levels were identified as having the potential to predict the risk of postoperative recurrence [69], thus supporting the utility of this non-invasive tool in predicting postoperative recurrence and facilitating timely and targeted interventions to enhance long-term outcomes for individuals with CRSwNP.

### 2.2. Allergic Rhinitis (AR)

AR is a common IgE-mediated inflammatory disease of the nasal mucosa, with an increasing global prevalence. It is clinically characterized by symptoms such as nasal obstruction, watery rhinorrhea, sneezing, and nasal itching [70]. Extensive evidence identifies type 2 inflammation as the primary pathophysiological mechanism of AR, explaining its strong pathophysiological, epidemiological, and clinical associations with asthma [71,72]. The pathogenesis of allergic rhinitis is associated with genetic, environmental, and epigenetic factors. In this context, research has highlighted that the abnormal expression of non-coding microRNAs is linked to the development of combined AR and asthma syndrome (CARAS) [39].

Panganiban et al. demonstrated that miRNAs expressed in the blood could serve as non-invasive biomarkers for diagnosing and characterizing both asthma and AR. Specifically, circulating levels of miR-125b, miR-16, miR-299-5p, miR-126, miR-206, and miR-133b were identified as the most predictive markers of allergic and asthmatic status. The authors concluded that specific subsets of circulating miRNAs are distinctly expressed in patients with AR and asthmatic patients, presenting potential as non-invasive diagnostic tools for these conditions [40].

Among miRNAs related to the immune system, miR-126 plays a key role in regulating inflammation and autoimmunity. It is abnormally expressed in allergic respiratory diseases, contributing to the onset and progression of AR, and shows a positive correlation with disease severity [73]. The inhibition of miR-126 expression has been shown to reduce eosinophil accumulation in the airway and suppress Th2-driven airway inflammation, airway hyperresponsiveness, and excessive mucus production [74].

Another study further supported miR-126’s involvement in AR pathogenesis by positively regulating the Treg cytokine FOXP3, while negatively regulating the Th1 and Th2 cytokines IFN-γ and IL-4 [75].

Likely, miR-155 is the most prominent regulator of immune function and has been extensively studied in allergic diseases [76]. Notably, miR-155 plays a pivotal role in both acute and chronic inflammatory responses [77]. It is involved in the local regulation of Th2 responses during allergen-induced eosinophilic airway inflammation, highlighting its potential as a therapeutic target for allergic inflammatory diseases [78]. Zhu et al. demonstrated that miR-155 significantly influences Th2 factor expression and the allergic inflammatory response in type-2 innate lymphoid cells (ILC2s) in AR. Their study showed that both miR-155 and ILC2s are overexpressed in AR patients. Moreover, the findings indicate a strong correlation between elevated ILC2 ratios and increased miR-155 expression. The overexpression of ILC2s contributes to the AR inflammatory response by producing large amounts of Th2 cytokines, including IL-4, IL-5, IL-9, and IL-13. These results suggest that targeting miR-155 and ILC2 expressions could represent a promising therapeutic approach for managing AR [41].

An interesting study by Liu et al. assessed the effect of miR-124-3p on type 2 inflammation in AR, finding that miR-124-3p may reduce type 2 inflammation by modulating IL-4Rα signaling. These findings suggest that miR-124-3p could serve as a novel potential target for AR treatment [42].

Long and Zhang demonstrated that upon allergen stimulation, human nasal epithelial cells significantly inhibit the release of inflammatory cytokines through the increased expression of miR-181a-5p, which inhibits p38 *MAPK* signaling activation by negatively regulating IL-33 [43].

Finally, Liu et al. emphasized that the onset and progression of both AR and CRSwNP are associated with miRNAs, which modulate the pathogenesis of these conditions by regulating intercellular communication [79].

## 3. miRNAs in Oncological Nasal Diseases

### 3.1. Benign Tumors

Sinonasal inverted papilloma is a benign epithelial tumor arising from the Schneiderian mucosa whose etiological factors include smoking, allergies, occupational exposures, and chronic inflammation [80].

However, the molecular genetic alterations and pathophysiological mechanisms underlying sinonasal inverted papilloma remain poorly understood. Dysregulation of miRNAs has been implicated in tumorigenesis, yet their role and clinicopathological significance in sinonasal inverted papilloma are not well defined. To date, only a few studies have explored the role of miRNAs in this benign neoplasm.

Kakizaki et al. proposed that miR-296-3p may play a critical role in the malignant transformation of sinonasal inverted papillomas by regulating *PTEN*, a tumor suppressor gene typically associated with human carcinogenesis [44]. Teng et al. identified that miR-214-3p expression was significantly decreased in sinonasal inverted papilloma tissues, correlating with both staging and recurrence of the disease. These findings suggest that miR-214-3p could serve as a biomarker and therapeutic target for this neoplasm [45].

Furthermore, Re et al. reported that miR-449 may contribute to the molecular pathogenesis of sinonasal inverted papilloma and its malignant transformation into sinonasal squamous cell carcinoma. As such, miR-449 could serve as a tumor biomarker and potentially as a diagnostic and monitoring tool for detecting and tracking malignant transformation in patients affected by sinonasal inverted papilloma [46].

Juvenile angiofibroma is also classified as a benign nasal tumor and represents a distinct fibrovascular neoplasm that primarily affects adolescent males. The lesion typically originates from the superior margin of the sphenopalatine foramen and is highly vascularized, with blood supply from branches of the external carotid artery, particularly the maxillary and sphenopalatine arteries, as well as the internal carotid artery. The most common symptoms include nasal obstruction and epistaxis. While juvenile angiofibroma is benign, it can exhibit an aggressive growth pattern, including bone destruction [81]. Although the clinical features are well established, the underlying biology of the tumor has only begun to be understood.

Research on miRNAs in juvenile angiofibroma remains limited. Lerner et al. investigated miRNA expression in juvenile angiofibroma and identified a statistically significant downregulation of miR-125a-5p. This downregulation likely stimulates tumor growth due to the loss of its tumor-suppressive function, a role that has been well documented in previous studies. Additionally, they found downregulation of miR-218 and its impact on *β-catenin*, emphasizing the importance of *β-catenin* in tumor biology [47].

### 3.2. Malignant Tumors

Primary malignancies of the nose and paranasal sinuses are rare, accounting for less than 0.5% of all cancers. The most common subtypes include epithelial tumors, such as sinonasal squamous cell carcinoma (SNSCC) and sinonasal adenocarcinoma (SNADC), followed by adenoid cystic carcinoma (AdCC), esthesioneuroblastoma (ENB), sinonasal neuroendocrine carcinoma (SNEC), sinonasal undifferentiated carcinoma (SNUC), and mucosal melanoma. In particular, among SNADCs, intestinal-type sinonasal adenocarcinoma (ITAC) is considered the most representative subtype. Exposure to factors like wood and leather dust, tobacco smoke, HPV, and certain occupational hazards are considered possible risk factors for these malignancies. However, the evidence remains unclear, and the exact causes and molecular mechanisms underlying the pathogenesis of these cancers are not fully understood [82].

Next-generation sequencing (NGS)-based miRNome analysis identified the miR-205 and miR-34c/miR-449 clusters as miRNA superfamilies involved in the pathogenesis of sinonasal cancers, particularly ITAC [48]. Research on this topic is advancing rapidly, and the latest study by Tomasetti et al. has provided evidence that miR-34/miR-449 and their target genes are deregulated in sinonasal cancers, suggesting that further studies are desirable to determine if they could serve as potential prognostic biomarkers [49].

The same authors also investigated the miR-126 expression levels in ITACs, comparing them to benign nasal lesions such as inflammatory nasal polyps and sinonasal inverted papillomas. They found that miR-126 could significantly differentiate malignant from benign nasal lesions. Previous studies have demonstrated that miR-126 suppresses tumor growth by inhibiting cancer cell migration, proliferation, and invasion. Tomasetti et al. observed a significant reduction in miR-126 levels in ITAC tissue, while the levels in benign lesions were markedly higher compared to that of matched normal mucosa. This suggests that circulating miR-126 could serve as a potential biomarker to distinguish malignant from benign nasal neoplastic conditions. The reduced miR-126 levels in ITACs highlight its role in cancer progression. Restoring miR-126 inhibited cell growth, induced metabolic changes, and reduced the tumorigenic potential of malignant cells [50].

As extensively demonstrated, dysregulated miRNAs play critical roles in the onset and progression of various tumors, including those affecting the nasal cavity and paranasal sinuses. In this field, the study by Qian et al. is of significant interest, as it identifies miR-143-3p as a potential molecular therapeutic target for SNSCC. The authors demonstrated that miR-143-3p, which is markedly under-expressed in SNSCC tissues and cell lines, can modulate the growth and migration of malignant cells by directly targeting *Bcl-2* and *IGF1R* [51].

Nohata et al. reported a significant reduction in the expression of miR-874, miR-1, and miR-133a in maxillary sinus SCC cells. Their study specifically highlighted the functional importance of these miRNAs, revealing that miR-874 acts as a tumor-suppressive miRNA by directly regulating PPP1CA, which may function as an oncogene. Furthermore, the restoration of downregulated miR-1 and miR-133a in malignant cells demonstrated that both miRNAs notably induced apoptosis and inhibited cancer cell proliferation. These findings suggest that the characterization of new miRNA-regulated cancer pathways could offer new knowledge of the molecular mechanisms underlying maxillary sinus SCC oncogenesis [52,53].

The study by Kinoshita et al. is of considerable importance, as it revealed a significant reduction in the expression of miR-375, along with an increased expression of lactate dehydrogenase B in malignant cells of maxillary sinus SCC. The authors demonstrated that miR-375 functions as a tumor suppressor and directly regulates lactate dehydrogenase B in this cancer. Restoring miR-375 notably inhibited malignant cell proliferation, further supporting its role as a tumor suppressor in maxillary sinus SCC [54]. Sun et al. also investigated the impact of miRNAs on the development and progression of maxillary sinus SCC, finding that the long noncoding RNA *KCNQ1OT1* could directly interact with miR-204. They demonstrated that the *KCNQ1OT1/miR-204* axis facilitates maxillary sinus SCC progression [83].

Finally, regarding sinonasal AdCC, Alerraqi et al. investigated the molecular characteristics of AdCC with various histological features across three distinct anatomical sites: the parotid gland, the paranasal sinuses, and the mammary gland. Their findings demonstrated that sinonasal hyalinizing AdCC is molecularly distinct from its salivary and breast counterparts, which may have implications for prognosis and treatment strategies [84].

## 4. miRNA-Based Therapy: An Approach for Sinonasal Cancer Treatment

As reported, miRNAs can either promote or inhibit tumor progression by modulating various signaling pathways, functioning as oncogenic miRNAs (oncomiRs) or tumor suppressors. Consequently, restoring the expression of downregulated tumor-suppressor miRNAs has emerged as a promising therapeutic strategy for more effectively inhibiting tumorigenesis. In this context, miR-126 is typically downregulated in tumors and primarily acts as a tumor suppressor by regulating specific gene networks, as demonstrated in glioma, non-small cell lung cancer, pancreatic cancer, colorectal cancer, breast cancer, and sinonasal cancer [50,85,86].

Beyond epigenetic mechanisms implicated in *EGFL7/miR-126* downregulation, chromatin remodeling has been identified as an alternative regulatory mechanism, leading to miR-126 upregulation in asbestos-induced inflammation while being suppressed in malignant cells [87]. Transcription factors (TFs) play a crucial role in miR-126 expression. Several TFs, including *KLF2* (*Krüppel-Like Factor 2*), *ETS2* (*ETS Proto-Oncogene 2*), and *EBF1* (*Early B Cell Factor 1*), are frequently deregulated in various cancers, including sinonasal cancer, and possess multiple putative binding sites on the *EGFL7* gene. Silencing these TFs significantly reduced pre-miR-126 expression, with KLF2 and EBF1 siRNAs demonstrating the strongest effect [88]. The same study indicated that *KLF2* regulates miR-126 expression both directly and indirectly by influencing *EBF1* and *ETS2* transcription. Deregulation of this regulatory axis may contribute to malignant transformation by altering the expression of target genes involved in key cancer-related pathways, including cell growth, adhesion, migration, and metastasis.

Restoring miR-126 expression resulted in a significant reduction in tumor cell proliferation in vitro and suppressed tumor formation in vivo, further underscoring its potential as a therapeutic target [86].

Exosomal transfer of miR-126 resulted in the dose-dependent inhibition of malignant nasal-septum carcinoma (MNSC) cell growth associated with the inhibition of its gene targets expression, such as *insulin receptor substrate-1* (*IRS1*) and *VEGF* [50]. The transfer of miR-126 mediated by exosomes presents a new approach to develop miRNA-based therapies for neoplastic pathologies as well as improved diagnostic tools.

Exosomes, as carriers of bioactive molecules, play a central role in facilitating communication within the tumor microenvironment. Their small size, stability, low toxicity and immunogenicity, and their capacity to cross biological barriers make them attractive candidates for targeted drug delivery systems.

In an in vivo study, exosomes encapsulating miR-126 were shown to inhibit NSCLC growth and metastasis, resulting in a reduced number of metastatic lung nodules in nude mouse models [89,90]. Additionally, miR-126 has been demonstrated to suppress pleural mesothelioma (PM) by targeting cancer-related genes without causing toxicity or histopathological alterations [91]. Exosomes enriched with miR-126 (exo-miR) efficiently penetrated PM-derived spheroids and induced cytotoxic effects in both in vitro and in vivo models. In particular, regarding the in vitro results, the cell lines used included the pleural mesothelioma cell lines H28 and MSTO-211H, obtained from ATCC [92].

**Figure 1:**
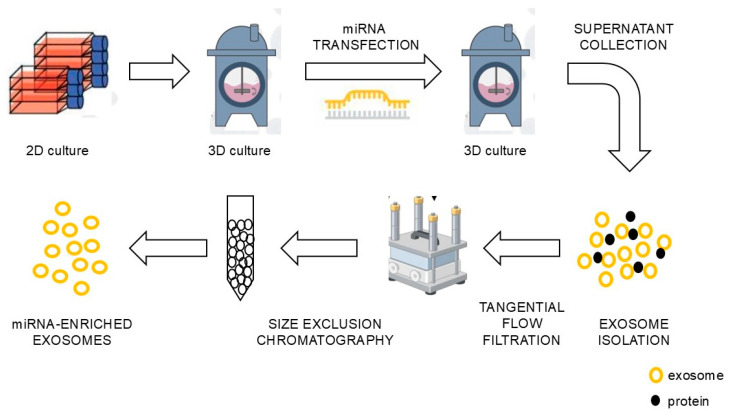
The production of exosome-carrying onco-miRNAs involves donor cells being cultured in cell flasks or bioreactors, loaded with onco-suppressor miRNA by transfection, isolated by ultrafiltration using tangential flow filtration (TFF), and purified through size-exclusion chromatography (SEC).

Although no exosome-based therapy has received FDA approval to date, the number of ongoing clinical trials investigating exosome-based therapeutics is steadily increasing, highlighting their significant potential to transform the treatment of various diseases, including cardiovascular conditions, cancer, and neurological disorders. The development of high-quality exosomes carrying tumor-suppressor miRNAs is critical for advancing cancer therapeutics. However, challenges remain regarding isolation methods, loading techniques, analytical characterization, and clinical implementation. Exosomes are highly abundant, can be derived from a diverse range of donor cells, and exhibit unique biological properties, making them promising candidates for innovative cancer treatment strategies. A schematic representation of exosome production for clinical applications is illustrated in Figure 1.

## 5. Conclusions

**Figure 2:**
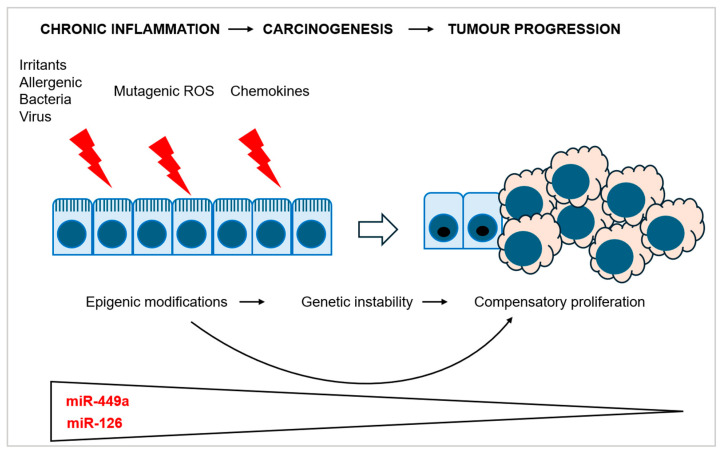
miRNA levels in chronic inflammation-induced sinonasal carcinogenesis. Chronic inflammation induced by environmental factors leads to epigenetic changes, resulting in an increase in miR-449a and miR-126 levels, which gradually decrease during malignant transformation. ROS: reactive oxygen species.

Chronic inflammation-induced carcinogenesis is a process that involves epigenetic modification driving cell transformation where miRNAs play a central role (Figure 2). A deeper understanding of the molecular oncogenic pathways involving miRNAs in both inflammatory and oncologic nasal diseases has the potential to significantly improve diagnostic accuracy, offering more reliable biomarkers for early detection. Moreover, this knowledge could lead to the development of more targeted and effective therapeutic strategies, including personalized treatment plans, and contribute to the advancement of effective disease prevention measures. Continued research in this area will be crucial to fully realize these potential benefits and improve patient outcomes.

## Figures and Tables

**Table 1:** Summary of the main findings of the original articles included in this review.

	Disease	Authors	Year	Country	Sample	Sample Size and Control	miRNA	Expression
Inflammatory Nasal Diseases	Chronic Rhinosinusitis	Silveira MLC et al. [23]	2021	Brazil	Tissue	36 CRSwNP 41 control	miR-205-5p	↑
		Song L et al. [24]	2022	China	Tissue	37 CRSwNP 29 CRSsNP	miR-125b	↑
		Yu J et al. [25]	2021	China	Tissue	3 ECRSwNP 3 nonECRSwNP 3 control	miR-132-3p; miR-145-5p; miR-146a-5p; miR-27b-3p	↓
		Xia G et al. [26]	2015	China	Tissue	40 CRS 5 control	miR-125b; miR-155; miR-146a	↑
							miR-92a; miR-26b; miR-181b	↓
		Zhang XH et al. [27]	2012	China	Tissue	43 CRSsNP 46 ECRSwNP 31 nonECRSwNP 50 control	miR-125b	↑
		Ma ZX et al. [28]	2015	China	Blood	30 CRS 7 control	miR-125b-5P; miR-150-5P; miR-210-3P	↑
							miR-708b-5P; miR-126-3P	↓
		Gata A et al. [29]	2023	Romania	Tissue	86 CRSwNP 20 control	miR-125b	↑
							miR-203a-3p	↓
		Du J et al. [30]	2020	China	Tissue	25 ECRSwNP 25 nonECRSwNP 25 CRSsNP 30 control	miR-155	↑
		Bu X et al. [31]	2021	China	Tissue	10 ECRSwNP 5 nonECRSwNP 9 control	miR-154; miR-221; miR-223; let-7; miR-34/449	↑
		Liu CC et al. [32]	2018	China	Tissue	20 CRSwNP 20 control	miR-124	↓
		Korde A et al. [33]	2020	USA	Serum/ Tissue	40 CRS	miR-1	↓
		Qing X et al. [34]	2021	China	Tissue	20 CRSwNP 20 control	miR-142-3p	↑
		Luo XQ et al. [35]	2017	China	Blood	26 CRSwNP 10 control	miR-19-a	↑
		Luan G et al. [36]	2022	China	Tissue	20 ECRSwNP 12 nonECRSwNP 16 control	miR-21-5p	↑
		Yang N et al. [37]	2020	China	Tissue	14 CRSsNP 11 CRSwNP 10 control	miR-155-5p	↑
		Li X et al. [38]	2019	China	Tissue	13 CRSwNP 12 CRSsNP 11 control	miR-21	↑
	Allergic Rhinitis	Liu Z et al. [39]	2024	China	Blood	38 CARAS 43 control	miR-4454	↑
		Panganiban RP et al. [40]	2016	USA	Blood	35 asthma 25 AR 19 control	miR-125b; miR-16; miR-299-5p; miR-126; miR-206; miR-133b	↑
		Zhu YQ et al. [41]	2019	China	Tissue	26 AR 28 control	miR-155	↑
		Liu Q et al. [42]	2022	China	Tissue	6 AR 6 control 6 agomir 6 antagomir	miR-124-3p	↓
		Long S et al. [43]	2021	China	Tissue	15 AR 15 control	miR-181a-5p	↓
Benign Nasal Tumors	Sinonasal Inverted Papilloma	Kakizaki T et al. [44]	2017	Japan	Tissue	5 SNIP 5 SNIP-SNSCC	miR-296-3p	↑ *
		Teng Y et al. [45]	2018	China	Tissue	32 SNIP 12 control	miR-214-3p	↓
		Re M et al. [46]	2021	Italy	Tissue	33 SNIP 17 SNIP-SNSCC	miR-449	↑
	Juvenile Angiofibroma	Lerner C et al. [47]	2014	Germany	Tissue	13 JA 3 control	miR-125a-5p; miR-218	↓
Malignant Nasal Tumors	ITAC	Re M et al. [48]	2022	Italy	Tissue	43 ITAC	miR-205; miR-34c/449a	↓
							miR-192	↑
		Tomasetti M et al. [49]	2024	Italy	Tissue	80 SNC	miR-34c; 449a	↓
		Tomasetti M et al. [50]	2018	Italy	Serum/Tissue	23 ITAC 15 SNIP 20 NIP	miR-126	↓
	SNSCC	Qian Y et al. [51]	2019	China	Tissue	12 SNSCC	miR-143-3p	↓
	Maxillary Sinus SCC	Nohata N et al. [52]	2011	Japan	Tissue	20 MSSCC	miR-874	↓
		Nohata N et al. [53]	2011	Japan	Tissue	20 MSSCC	miR-1; miR-133a	↓
		Kinoshita T et al. [54]	2012	Japan	Tissue	20 MSSSCC	miR-375	↓

Legend. CRS: Chronic Rhinosinusitis; AR: Allergic Rhinitis; CRSwNP: Chronic Rhinosinusitis with Nasal Polyps; CRSsNP: Chronic Rhinosinusitis without Nasal Polyps; ECRSwNP: Eosinophilic Chronic Rhinosinusitis with Nasal Polyps; CARAS: Combined AR and Asthma Syndrome; SNIP: Sinonasal Inverted Papilloma; SNSCC: Sinonasal Squamous Cell Carcinoma; SCC: Squamous Cell Carcinoma; SNIP-SNSCC: SNSCC arousing from SNIP; JA: Juvenile Angiofibroma; SNC: Sinonasal Cancer; NIP: Nasal Inflammatory Polyps; MSSCC: Maxillary Sinus SCC; * in SNIP-SNSCC.

## Data Availability

No new data were created or analyzed in this study. Data sharing is not applicable to this article.

## Abbreviations

miRNAs	microRNAs
MVB	multivesicular body
CRS	chronic rhinosinusitis
AR	allergic rhinitis
SNSCC	sinonasal squamous cell carcinoma
EPOS	European Position Paper on Rhinosinusitis and Nasal Polyps
HRQoL	health-related quality of life
CRSwNP	chronic rhinosinusitis with nasal polyps
CRSsNP	chronic rhinosinusitis without nasal polyps
IL	interleukin
TNF-α	tumor necrosis factor α
EMT	epithelial–mesenchymal transition
CARAS	combined allergic rhinitis and asthma syndrome
Treg Th	regulatory T cells T helper cell
FOXP3	forkhead box P3
IFN-γ	interferon γ
HPV	human papillomavirus
SNADC	sinonasal adenocarcinoma
SNEC	sinonasal neuroendocrine carcinoma
SNUC	sinonasal undifferentiated carcinoma
ITAC	intestinal-type sinonasal adenocarcinoma
NGS	next-generation sequencing
TFs	transcriptional factors
KLF2	Krüppel-like factor 2
ETS2	ETS proto-oncogene 2
EBF1	early B cell factor 1
IRS1	insulin receptor substrate-1
VEGF	vascular endothelial growth factor
FDA	Food and Drug Administration
NSCLC	non-small cell lung cancer
